# Clinical profile of cluster headaches in China – a clinic-based study

**DOI:** 10.1186/1129-2377-14-27

**Published:** 2013-03-21

**Authors:** Zhao Dong, Hai Di, Wei Dai, Meiyan Pan, Zheng Li, Jingyao Liang, Mingjie Zhang, Zhibin Zhou, Ruozhuo Liu, Shengyuan Yu

**Affiliations:** 1 International Headache Center, Department of Neurology, Chinese PLA General Hospital, Fuxing Road 28 Haidian District, Beijing 100853, China

**Keywords:** Cluster headache, Chinese, Features, Sense of restlessness

## Abstract

**Background:**

The clinical profile of cluster headache in Chinese patients have not been fully studied.

**Methods:**

The classification and clinical features of 120 consecutive patients with cluster headache (105 males, 15 females; mean age, 34.9 ± 10.5 years) visiting at International Headache Center from May 2010 to August 2012 were analyzed.

**Results:**

Patients came from 16 different regions of China. Mean age at onset of cluster headache was 26.7 ± 10.9 years. Only 13 patients (10.8%) had previously been diagnosed with cluster headache. Mean time to diagnosis from first symptoms was 8.2 ± 7.1 years (range, 0–35 years). Chronic cluster headache was observed in only 9 patients (7.5%). The most commonly reported location of cluster headache was temporal region (75.0%), followed by retro-orbital region (68.3%), forehead (32.5%), vertex (32.5%) and occipital (22.5%). Lacrimation was the most consistently reported autonomic feature (72.5%). During acute attacks, 60.0% of patients experienced nausea, and 41.7% experienced photophobia and 40.8% experienced phonophobia. In addition, 38.3% reported restless behavior and 45.8% reported that physical activity exacerbated the pain. None of patients experienced visual or other kinds of aura symptoms before cluster attacks. We found that 38.3% of patients had <1 cluster period and 35.8% for 1–2 cluster periods per year with these periods occurring less frequently during the summer than during other seasons. Cluster duration was 1–2 months in 32.5% of patients. During cluster periods, 73.3% of patients had 1–2 attacks per day, and 39.2% experienced cluster attacks ranging in duration from 1 h to less than 2 h. The duration of attacks were 1.5 (1–2.25) hours for males and 1.5 (1-3) for females respectively. The World Health Organization quality of life-8 questionnaire showed that cluster headache reduced life quality.

**Conclusions:**

Compared to Western patients, Chinese patients showed a relatively low prevalence of chronic cluster headaches, pain sites mainly focused on areas distributed by the first division of the trigeminal nerve, a low frequency of restlessness and absent aura. These clinical features may be more common in Eastern populations, including mainland Chinese, Japanese and Taiwanese patients, than in Western patients.

## Background

Cluster headache (CH) is an excruciating primary headache disorder, classified with similar conditions known as trigeminal autonomic cephalalgias [1]. Patients always describe the pain of a single attack as being worse than anything else they have experienced. Headaches are characterized by unilateral pain usually involving the orbital or periorbital region innervated by the first (ophthalmic) division of the trigeminal nerve and are accompanied by ipsilateral autonomic features, including lacrimation, conjunctival injection, nasal congestion and/or rhinorrhea, ptosis and/or miosis, and periorbital edema. CH show male predominance and a periodic occurrence and circadian rhythm of cluster attacks [2-4]. Diagnostic criteria for CH have been established by the International Headache Society [1]. Knowledge and understanding of CH derive primarily from studies in Western populations [5-13]. The clinical characteristics of CH in other regions of the world, including Asia, however, are not well understood [14,15]. To our knowledge, little is known about the characteristics of CH in patients from the Chinese mainland. We have characterized the clinical profile of CH in China by surveying CH patients registered at a headache clinic in Beijing, China.

## Methods

The study population consisted of patients diagnosed with CH, as defined by the second edition of the International Classification of Headache Disorders (ICHD-II) [1], on first consultation from May 2010 to August 2012 at the International Headache Center of Chinese PLA General Hospital in Beijing, China, accredited by International Headache Society. For each patient, a detailed clinic questionnaire for headache disorders was completed by a certified neurologist in headache center during the initial consultation and the diagnosis was made by at least two headache specialists together. Magnetic resonance imaging of the head/brain was applied to rule out symptomatic origin for every headache patient.

A detailed database for each headache patient was set up including clinical information such as age, gender, course of disease, pain intensity, possible trigger factors, autonomic features, additional features (e.g. nausea, vomiting, photophobia, phonophobia, behaviors during attacks, and aggravation after activity), frequency and duration of clusters, frequency and duration of attack onset, family history of headache, history of smoking and drinking, and quality of life. Maximum pain intensity was estimated using a visual analogue scale (VAS). The study protocol was approved by the ethics committee of the Chinese PLA General Hospital, Beijing.

All measurements were reported as mean ± SD. Categorical variables were compared using the chi-square test, and continuous variables were compared using Student’s *t*-test or one-way analysis of variance (ANOVA). SPSS for Windows, Version 20.0, was used for statistical analyses with the significance level set at *P =* 0.05.

## Results

### Area distribution of patients

**Figure 1:**
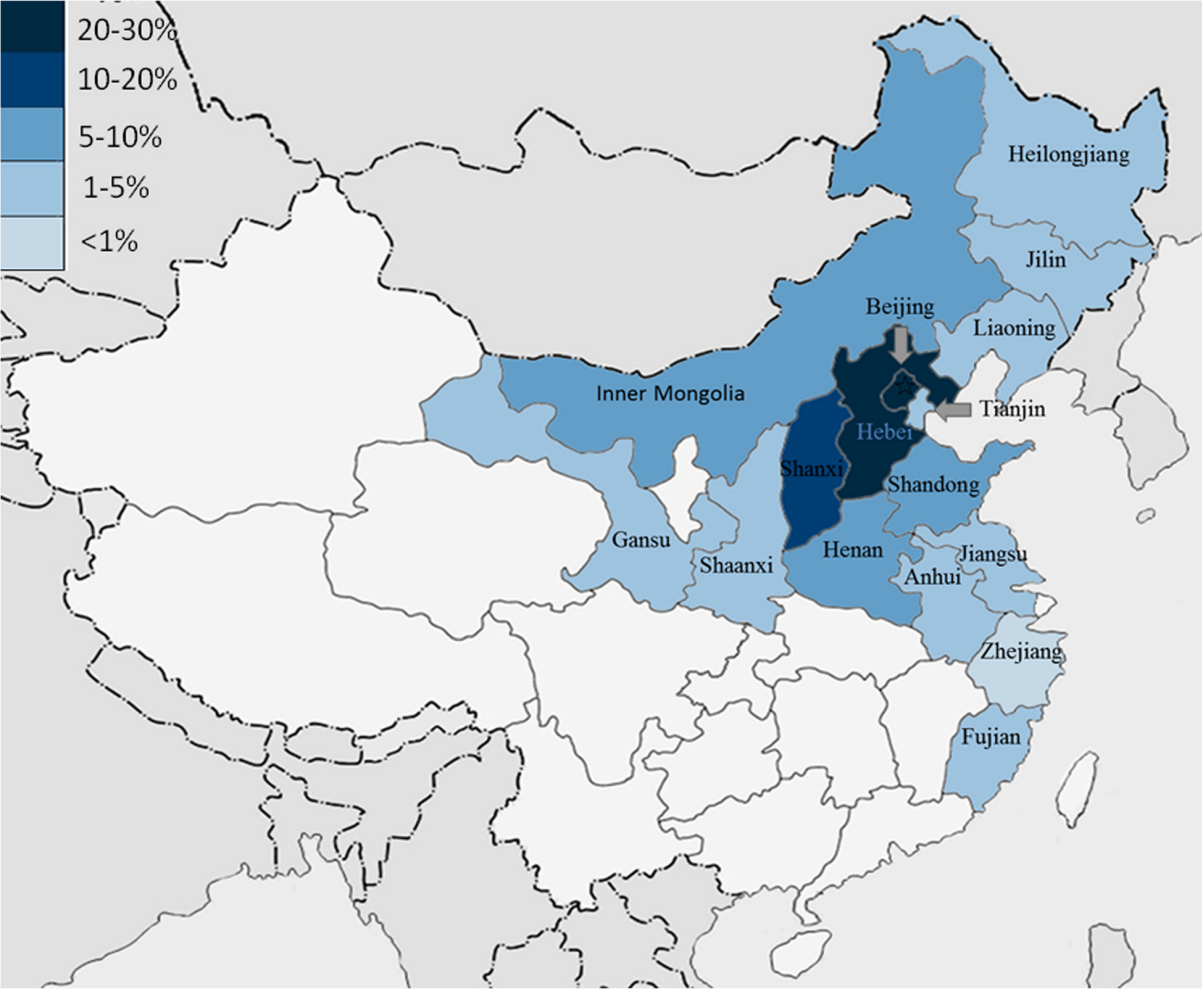
**Regional distribution of cluster headache patients in the current study (n = 120).** Patients came from 16 regions of China. Colors from dark to light indicate different proportion of headache patients from higher to lower (5 levels: 20–30%, 10–20%, 5–10%, 1–5%, and <1%).

Patients came from 16 regions of China (Figure 1). Most of them lived in North and Eastern China including Hebei (29, 24.2%), Beijing (25, 20.8%), Shanxi (15, 12.5%), Inner Mongolia (8, 6.7%), Henan (8, 6.7%), Shandong (10, 8.3%). Other areas consist of Anhui (4, 3.3%), Gansu (3, 2.5%), Heilongjiang (2, 1.7%), Jilin (3, 2.5%), Jiangsu (3, 2.5%), Fujian (2, 1.7%), Liaoning (3, 2.5%), Shaanxi (2, 1.7%), Tianjin (2, 1.7%) and Zhejiang (1, 0.8%).

### Study population

**Figure 2:**
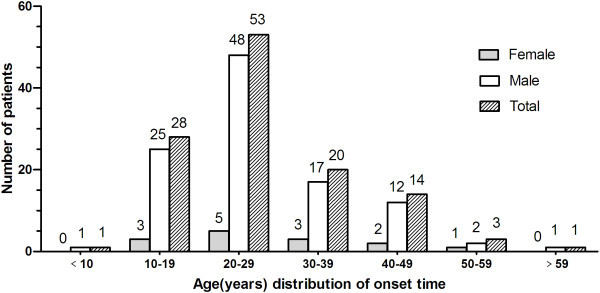
**Age distribution at onset for cluster headache.** The peak age at onset for male and female were both in the 2^nd^ decade of life.

Of the 120 patients enrolled in this study, 105 were male and 15 were female, giving a male-to-female (M: F) ratio of 7:1; of these, 111 (92.5%) had episodic CH (ECH) and 9 (7.5%) had chronic CH (CCH). Mean age at first consultation at our clinic was 34.9 ± 10.5 years (males, 34.7 ± 10.6 years vs. females, 36.5 ± 9.7 years). Mean age at onset was 26.7 ± 10.9 years and was similar in males and females (26.6 ± 10.8 years vs. 28.0 ± 11.4 years, P = 0.652). The mean VAS of all patients was 8.9 ± 1.4, 8.9 ± 1.4 in males and 8.6 ± 1.6 in females (P = 0.454) (Table 1). One CH male patient has already suffered trigeminal neuralgia. Two patients (1.6%, 2 M) also had migraine headaches and five patients (4.2%, 3 M, 2 F) had tension-type headaches. Eight patients (6.7%) gave a family history of CH (diagnosed with identified and classic clinical features), including mother (two patients), father (four patients) and other relatives (two patients). Peak age at onset was 20–29 years for both males and females (Figure 2).

**Table 1:** Demographics of subjects with cluster headache

**Patient characteristics**	**Total N = 120**	**Males n = 105**	**Females n = 15**	**P-value**
Type of cluster headache				
Episodic	111	97	14	1.00
Chronic	9	8	1	
Age in years (mean ± SD)	34.9 ± 10.5	34.7 ±10.6	36.5 ± 9.7	
Age at onset in years (mean ± SD)	26.7 ± 10.9	26.6 ± 10.8	28.0 ± 11.4	0.652
Pain intensity (VAS)	8.9 ± 1.4	8.9 ± 1.4	8.6 ± 1.6	0.454
Family history of Cluster headache	8			
Mother	2			
Father	4			
Others	2			

*The ratio of males to female was 7:1.

### History of smoking and drinking

Sixty-six (55%) CH patients had a positive history of tobacco exposure including 59 (49.2%) current smokers and 7 (5.8%) ex-smokers. Other 45% (54/120) sufferers stated they had never smoked prior to cluster headache onset. Almost 49.2% (59/120) of the surveyed patients stated they drank alcohol and 5% (6/120) have stopped drinking.

### Sites and laterality of headache

The most commonly reported location of CH was temporal region (75.0%), followed by retro-orbital region (68.3%), forehead (32.5%), vertex (32.5%) and occipital (22.5%) (Table 2). Other sites of pain included neck (4.2%), teeth (4.2%), ear (4.2%), cheek (2.5%) and nose (1.7%). Strictly unilateral headache was most frequently reported (right side 51.7%, left side 36.7%), followed by predominantly right side (4.2%) and left side (3.3%). However, six (5.0%) patients also experienced equal attacks on shifting sides among different attacks.

**Table 2:** The locations and laterality of pain in CH patients

	**Total (N = 120)**	**Males (n = 105)**	**Females (n = 15)**	**P-value**
**Sites of pain**				
Temporal	90 (75.0)	78 (74.3)	12 (80.0)	0.759
Retro-orbital	82 (68.3)	71 (67.6)	11 (73.3)	0.773
Forehead	39 (32.5)	36 (34.3)	3 (20.0)	0.381
Vertex	39 (32.5)	32 (30.5)	7 (46.7)	0.244
Occipital	27 (22.5)	24 (22.9)	3 (20.0)	1.00
Neck	5 (4.2)	5 (4.8)	0	1.00
Teeth	5 (4.2)	5 (4.8)	0	1.00
Ear	5 (4.2)	5 (4.8)	0	1.00
Cheek	3 (2.5)	3 (2.9)	0	1.00
Nose	2 (1.7)	2 (1.9)	0	1.00
**Laterality of pain**				
Right-side only	62 (51.7)	55 (52.4)	7 (46.7)	0.785
Left-side only	43 (35.8)	40 (38.1)	3 (20.0)	0.251
Predominant right-side	5 (4.2)	4 (3.8)	1 (6.7)	0.493
Predominant left-side	4 (3.3)	2 (1.9)	2 (13.3)	0.076
Changing sides	6 (5.0)	4 (3.8)	2 (13.3)	0.163

Results are reported as number of patients (percent).

### Cranial autonomic and additional features in patients with CH

Lacrimation (72.5%) was the most consistently reported autonomic feature, followed by conjunctival injection (63.3%), rhinorrhea (33.3%), nasal congestion (32.5%), and less commonly, blepharoedema (23.3%), facial sweating (18.3%), Ptosis/miosis (16.7%) (Table 3). Although cranial autonomic symptoms (CAS) of CH commonly occurred unilaterally, bilateral CAS including lacrimation (2/87), rhinorrhea (2/40), and facial sweating (3/22) were also observed in our cohort. During acute attacks, 60.0% of individuals experienced nausea, 41.7% reported photophobia, and 40.8% experienced phonophobia. In addition, 38.3% of patients experienced restless behavior, and 45.8% reported that physical activity exacerbated their pain. None of patients experienced visual or other kinds of aura symptoms before cluster attacks. There was no statistically significant difference in any of the clinical characteristics between male and female patient (Tables 2 and 3).

**Table 3:** Cranial autonomic and additional features in patients with cluster headache

	**Total (N = 120)**	**Males (N = 105)**	**Females (N = 15)**	**P-value**
**Autonomic features**				
Lacrimation	87(72.5)※	77(73.3)	10(66.7)	0.553
Conjunctival injection	76(63.3)	67(63.8)	9(60.0)	0.781
Rhinorrhea	40(33.3)※	38(36.2)	2(13.3)	0.140
Nasal congestion	39(32.5)	37(35.2)	2(13.3)	0.139
Ptosis/miosis	20(16.7)	17(16.2)	3(20.0)	0.714
Facial sweating	22(18.3)#	20(19.0)	2(13.3)	0.736
Blepharoedema	28(23.3)	23(21.9)	5(33.3)	0.338
**Additional features**				
Nausea	72(60.0)	64(61.0)	8(53.3)	0.585
Vomiting	40(33.3)	36(34.3)	4(26.7)	0.771
Photophobia	50(41.7)	43(41.0)	7(46.7)	0.782
Phonophobia	49(40.8)	42(40.0)	7(46.7)	0.780
Sense of restlessness and agitation	46(38.3)	40(33.3)	6(40.0)	1.00
Aggravation by physical activities	55(45.8)	46(43.8)	9(60.0)	0.277
**Aura**	0	0	0	

Results are reported as number of patients (percent). ※ The symptom occurred bilaterally in two patients. # The symptom occurred bilaterally in three patients.

### Periodicity of CH

**Figure 3:**
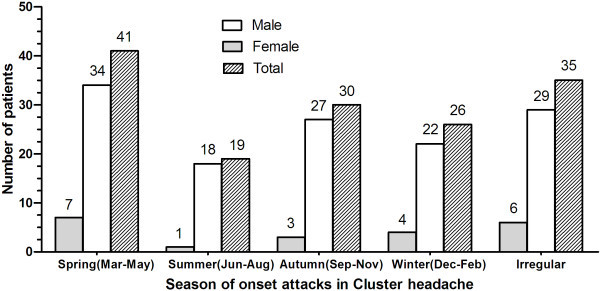
**Season of onset attacks in cluster headache.** A seasonal propensity of bout onset occurred mostly in spring and less frequent during the summer months.

We found that 38.3% of patients had <1 cluster period and 35.8% for 1–2 cluster periods per year respectively. Only nine (7.5%) had > 2 cluster periods per year and 11.7% of patients have the first experience of cluster (Table 4). Almost 70.8% of individuals (85 patients) commented on a seasonal propensity of bout onset. This occurred mostly in spring (41/120, 34.2%). However, these periods were less frequent during the summer months (19/120, 15.8%) than during other two seasons including autumn (30/120, 25.0%) and winter (26/120, 21.7%) (Figure 3). The duration of cluster attacks were 1–2 months in 32.5% of patients, 2 weeks to less than 1 month in 28.3%, less than 2 weeks in 14.2% and more than 2 months in 6.7% (Table 4).

**Table 4:** Frequency and duration of clusters and onset of attacks

	**Total (N = 120)**	**Males (n = 105)**	**Females (n = 15)**
**Frequency of clusters**			
More than 2 times/yr	9 (7.5)	7 (6.7)	2 (13.3)
1-2 times/yr	43 (35.8)	36 (34.3)	7 (46.7)
Less than 1 time/yr	46 (38.3)	42 (40.0)	4 (26.7)
First experience of cluster	14 (11.7)	13 (12.4)	1 (6.7)
Irregular	8 (6.7)	7 (6.7)	1 (6.7)
**Duration of clusters**			
Less than 2 weeks	17 (14.2)	14 (13.3)	3 (20.0)
From 2 weeks to less than 1 month	34 (28.3)	27 (25.7)	7 (46.7)
From 1 to 2 months	39 (32.5)	36 (34.3)	3 (20.0)
More than 2 months	8 (6.7)	8 (7.6)	0
First experience of cluster	14 (11.7)	13 (12.4)	1 (6.7)
Irregular	8 (6.7)	7 (6.7)	1 (6.7)
**Frequency of onset of attacks**			
More than 2 times/day	16 (13.3)	12 (11.4)	4 (26.7)
1 to 2 times/day	88 (73.3)	81 (77.1)	7 (46.7)
Less than 1 time/day	16 (13.3)	12 (11.4)	4 (26.7)
**Duration of onset of attacks**			
Less than 1 h	21 (17.5)	18 (17.1)	3 (20.0)
From 1 h to less than 2 h	47 (39.2)	42 (40.0)	5 (33.3)
From 2 h to 3 h	41 (34.2)	36 (34.3)	5 (33.3)
More than 3 h	11 (9.2)	9 (8.6)	2 (13.3)

Results are reported as number of patients (percent).

**Figure 4:**
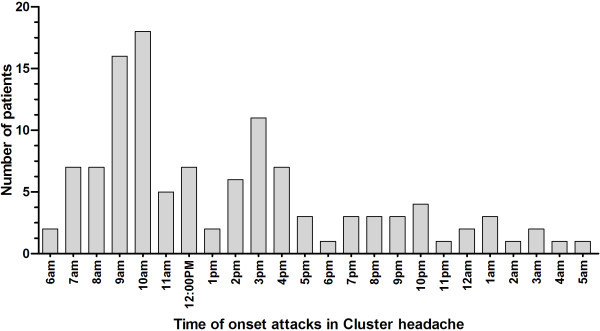
**Time of onset attacks in cluster headache.** Headaches occurred more commonly from 7 am to 10 am and from 2 pm to 4 pm.

**Figure 5:**
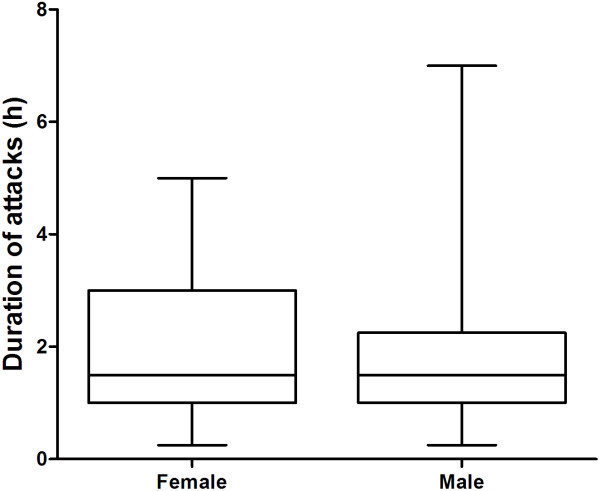
**Duration of attacks in cluster headache.** The duration of attacks were 1.5 (1–2.25) hours for males and 1.5 (1-3) for females respectively (P = 0.923).

Attacks occurred 1–2 times per day in 73.3% of patients, >2 times and <1 time per day both in 13.3% (Table 4). Eighty-one (67.5%) patients reported that their headaches occurred at a fixed time, more commonly from 7 am to 10 am (40%, 48/120) and from 2 pm to 4 pm (20%, 24/120) (Figure 4). There are also 39 patients (32.5%) who complained irregular headache attacks per day. We found that cluster attacks ranged in duration from 1 h to less than 2 h in 39.2% and from 2 h to 3 h in 34.2% of patients. Other 17.5% comment on the attack duration less than 1 h and 9.2% more than 3 h (Table 4). The duration of attacks were 1.5 (1–2.25) hours for males and 1.5 (1-3) for females respectively (P = 0.923) (Figure 5).

### Possible trigger factors

Thirty-three of 59 (55.9%) patients who had consumed alcohol reported headaches after alcohol consumption. Thirty-one (25.8%) patients felt that weather or temperature changes triggered their headache. Twenty-six (21.7%) patients experienced a headache attack when they had insufficient sleep and sixteen (13.3%) patients aggravated after fatigue. Fourteen (11.7%) patients complained of headaches after stress or labile mood. Finally, 4 patients reported that some special substances could induce headache attack (2 for cayenne pepper, 1 for gourmet power and 1 for stimulatory odour).

### Quality of life for CH patients

CH had a negative impact on Quality of life (QoL) (Table 5). Average scores about the eight items of WHOQoL-8 in people with CH including life quality, health level, daily life ability, satisfied with yourself, interpersonal relationship, habitation condition, daily life energy and payment ability were 2.38 ± 0.81, 2.08 ± 0.84, 2.68 ± 0.65, 2.57 ± 0.64, 2.85 ± 0.58, 2.73 ± 0.58, 2.09 ± 0.77 and 2.10 ± 0.60, respectively. It seems that the scores were lower than the data of migraine from a nationwide population-based headache survey in the mainland of China (Table 5) [16].

**Table 5:** World Health Organization quality of life-8 mean scores (SD) for cluster headache

	**Cluster headache (N = 120)**	**Migraine (N = 464)**[16]
Life quality	2.38 (0.81)	3.22 (0.67)
Health level	2.08 (0.84)	2.98 (0.83)
Daily life ability	2.68 (0.65)	3.35 (0.78)
Satisfied with yourself	2.57 (0.64)	3.45 (0.80)
Interpersonal relationship	2.85 (0.58)	3.76 (0.68)
Habitation condition	2.73 (0.58)	3.34 (0.84)
Daily life energy	2.09 (0.77)	3.03 (0.72)
Payment ability	2.10 (0.60)	2.60 (0.66)

04 of each item represents 5 grades of living quality from good to bad.

### Time delay for correct diagnosis

Only 13 patients (10.8%) had previously been diagnosed with CH. Mean time to diagnosis from first symptoms was 8.2 ± 7.1 years (range, 0–35 years). In 40% (48/120) of the CH patients, it took 10 years (14.2%) or longer (25.8%) to receive a correct diagnosis (Table 6). Only the minority of patients (10.8%) had a proper diagnosis of cluster headache in less than 1 year from symptom onset.

**Table 6:** Time delay for correct diagnosis of cluster headache

**Time delay to diagnosis**	**N (%)**
Less than 1 year	13 (10.8)
1 year	2 (1.7)
2 years	10 (8.3)
3 years	10 (8.3)
4 years	10 (8.3)
5 years	10 (8.3)
6 years	6 (5.0)
7 years	4 (3.3)
8 years	5 (4.2)
9 years	2 (1.7)
10 years	17 (14.2)
More than 10 years	31 (25.8)

Results are reported as number of patients (percent).

## Discussion

To our knowledge, this study is the first survey of a clinic-based sample of patients with CH in mainland China. Most clinical characteristics of CH reported in this study are consistent with other studies from Western and Eastern regions in the world, which included gender dominance for male [4-15,17-22], similar age at onset [5-7,9,13-15,18,19], temporal or retro-orbital regions as common sites of pain [5,7,13-15], lacrimation as the most frequent autonomic feature [5,7,13-15], high frequencies of migrainous features, low frequency of positive family history and seasonal propensity in spring and autumn (Table 7). Yet, it is also worth noting some discrepancies between the current and previous results.

**Table 7:** Clinical characteristics of CH from different regions in the world

	**UK**[5]**2002**	**Germany **[6]**2006**	**Germany**[7]**2012**	**Italy**[9]**2005**	**USA**[13]**2012**	**Japan**[15]**2011**	**Taiwan**[14]**2004**	**Mainland China**
**M:F Ratio**	2.5:1	3.5:1	3.4 :1	1.3:1	2.6:1	3.8: 1	6.4: 1	7:1
**CCH (%)**	21%	16.7%	31.1%	19%	--	3.5%	0	7.5%
**Mean age at onset (yrs)**	28.4 (ECH) 37 (CCH)	--	31.6	35.7	21-30^※ ^	31.0	26.9	26.7
**Family history of CH**	5%	--	--	--	18%	--	5.8%	6.7%
**Common sites of pain**	Retro-orbital, temporal, upper teeth	--	Peri-orbital, occipital, orofacial	--	Retro-orbital, upper teeth, jaw	Retro-orbital, temporal, occipital	Temporal, retro-orbital, occipital	Temporal, retro-orbital, forehead
**Predominant laterality**	Right	--	--	--	Right	Right	Right	Right
**Most cranial autonomic features**	Lacrimation (91%)	--	Conjunctival injection and/or lacrimation	--	Lacrimation (91%)	Lacrimation (66.3%)	Lacrimation (83%)	Lacrimation (72.5%)
**Most additional features**	Photophobia (56%)	Photophobia/Phonophobia (61.2%)	Photophobia/Phonophobia (73.2%)	--	Photophobia (48%)	Nausea (39.5%)	Phonophobia (58%)	Nausea (60.0)%
**Sense of agitation or restlessness**	93%	67.9%	83%	--	99.2%	69.8%	51%	38.3%
**Aura**	14%	23%	--	--	21%	--	1%	0
**Most common duration of attacks**	72-159 min	45-180 min (67.9%)	98 ± 75	--	--	From 1 h to less than 2 h (46.5%)	From 1 h to less than 2 h (34%)	From 1 h to less than 2 h (39.2%)
**Most common attack time**	Nocturnally (73%)	--	Nocturnally	--	Between 12 am and 3 am	Nocturnally(47.7%)	Midnight (28%), afternoon (27%)	Between 7 am and 10 am, 2 pm and 4 pm
**Seasonal propensity**	Spring and Autumn	--	Spring	--	Oct., Sep., Apr., Mar. and Nov.	--	Dec., Mar.	Spring

CH: cluster headache; CCH: chronic cluster headache; ECH: episodic cluster headache; ※: Peak age onset (36%).

In current study the CH was much more prevalent in men than in women, with an M: F ratio of 7:1. This data was similar to findings in Taiwan (6.4: 1) [14], but a little higher than other reported M: F ratios ranging from 1.3:1 to 3.8:1 [5-7,9,13,15]. Manzoni [20,21] have observed a time-related decrease in CH male predominance over the years and speculated that lifestyle may play an important role in the development of CH. Therefore, it may be conceivable that people from mainland China and Taiwan have similar M: F ratio due to their approximation of the lifestyle and cultural factors.

Of our 120 patients, only 9 (7.5%) had CCH. Other studies in Asian subjects have also reported a low prevalence of CCH (0–3.5%) [14,15]. This ratio is relatively much higher, however, in Western populations, in which 16.7–31.1% of patients with CH have been diagnosed with CCH [5-7,9]. The lower prevalence of CCH in Asian patients may be due to racial, lifestyle or cultural factors.

Temporal or retro-orbital regions were predominant sites of pain in CH patients, under the distribution of the first division of the trigeminal nerve (Table 7). Other areas such as upper teeth, jaw and maxilla were also very common in Western populations [5,7,13,23]. However, the pain of CH patients in current study was mainly focused on areas distributed by the first division of the trigeminal nerve and rarely on sites dominated by the second and third division of the trigeminal nerve (Table 2). This clinical feature was also very common in other Eastern patients, including Japanese and Taiwanese patients (Table 7).

Aura phenomena, similar to those experienced during migraine including visual and sensory phenomena, have been found to precede attacks in 5.9% to 21% of Western CH patients (Table 7) [5,6,13,23-25], which is the same prevalence of aura in migraine sufferers. This symptom appears to occur in both male and female patients with CH and in both chronic and episodic CH. None of our patients, however, were found to experience auras before cluster attacks. Similarly, only 1% of patients from Taiwan experienced aura [14]. The difference between Western and Eastern CH suffers may be also due to racial and genetic factors.

Studies in Western patients showed that 67.9% to 99.2% experience a sense of restlessness and agitation during an attack [5-7,13]. In contrast, we found that only 38.3% of our CH patients experienced restless and agitation. This finding is in agreement with results in other Asian populations, in that 51% of patients from Taiwan [14] and 69.8% of patients from Japan [15]. This discrepancy between Eastern and Western CH patients may be due to ethnic, social and/or cultural factors. We also found that 45.8% of headaches were aggravated by physical activities, a percentage higher than in Caucasian (21.7%) [6] and Taiwanese (7%) [14] patients, but similar to that of patients in Japan (31.0%) [15].

The signature feature of CH is its rhythmicity, which uniquely displays both a circannual and circadian periodicity [2-4]. We have observed a seasonal propensity of CH, with more attacks occurring in the spring and fewer during the summer than at other times of the year, a result consistent with previous findings [5,7,13,14]. The periodicity of CH suggests the involvement of the suprachiasmatic nucleus (SCN) of the hypothalamus, the biological clock [26-31]. Marked seasonal variations have been observed in the volume, total cell number and number of vasopressin expressing cells of the human SCN, with the SCN being smaller during the summer than during any other season of the year [32]. This may explain, at least in part, the lower prevalence of attack during the summer months in current study. We also found CH commonly occurred from 7 am to 10 am and from 2 pm to 4 pm compared to other time. However, we didn’t demonstrate most of the headache attacks occurred nocturnally as previous reports [5,7,13,15]. This discrepancy in circadian rhythmicity of CH attacks might be due to insignificant diurnal variations in the volume or vasopressin cell number of the human SCN in contrast with the annual cycle of the SCN [32].

Recent results from the United States Cluster Headache survey have revealed the differences between female and male CH including age of onset, family history, comorbid conditions, aura symptoms, pain locations and associated symptoms [33]. The data supported a previous study from a tertiary headache centre, which found some different characteristics in women with CH [22]. However, the current study showed no statistically significant difference in any of the clinical characteristics between male and female patient. The small sample size of our female patient population may result in such limit.

In the study we applied World Health Organization Quality of Life-8 (WHOQoL-8) to evaluate the quality of life in CH patients. This rating scale has also been widely used in a nationwide population-based headache survey in the mainland of China [16]. It seems that the scores of 8 items in CH patients were significantly lower than the data in migraineurs from above-mentioned survey. This may indicated CH had a more negative impact on quality of life than migraine.

Only 10.8% of our patients had previously been diagnosed with CH, a lower percentage than in other Asian countries, including Japan (14%). It took 10 years or even longer to receive a correct diagnosis for most of the CH patients in the study. These findings suggest that CH often remains unrecognized or misdiagnosed in China and that physicians may be unaware of this condition. Educating physicians about this recognizable and treatable condition and its diagnosis should be addressed.

This study had several limitations. Firstly, the clinical features were collected retrospectively and that this may result in a recall bias as compared to a prospective data collection with diaries. However, CH is a severe and excruciating headache disorder and thus the majority of the patients were easily to recall the clinical information about attacks. These may reduce the bias during interview. Moreover, the study has a relatively small number of CH patients, especially for female suffers. Lastly, patients were enrolled from a single headache clinic, although they came from 16 different regions in China.

## Conclusion

In summary, this study is the first to describe the clinical characteristics of CH in Chinese patients based on a clinic sample. Most of the clinical characteristic of these patients were consistent with results in other Asian and in Western patients, including similar age at onset, male predominance, temporal or retro-orbital regions as common sites of pain, similar pain intensity of primary headaches, lacrimation as the most frequent autonomic symptom, high frequencies of migrainous features, low frequency of positive family history and seasonal propensity in spring and autumn. We found that several characteristics were similar to those of other Asian populations, but differed from results in Western patients, including the low percentage of patients with chronic CH, pain sites mainly focused on areas distributed by the first division of the trigeminal nerve, the relative low frequency of restlessness and absent aura before headache attack. These may be due to different lifestyle, genetic, racial and cultural factors between Eastern and Western CH patients.

## Competing interests

The authors declared no conflict of interest.

## Authors’ contributions

SY ZD conceived and designed the experiments. ZD HD WD MP ZL JL MZ ZZ RL performed the experiments. ZD SY analyzed the data and drafted the paper. All authors read and approved the final manuscript.

## References

[B1] Headache Classification Subcommittee of the International Headache Society The International Classification of Headache Disorders: 2nd edition Cephalalgia 2004 14 Suppl 1 60 63 10.1111/j.1468-2982.2003.00824.x 14979299

[B2] May A Cluster headache: pathogenesis, diagnosis, and management Lancet 2005 14 843 855 10.1016/S0140-6736(05)67217-0 16139660

[B3] Nesbitt AD Goadsby PJ Cluster headache BMJ 2012 14 e2407 10.1136/bmj.e2407 22496300

[B4] Dodick DW Rozen TD Goadsby PJ Silberstein SD Cluster headache Cephalalgia 2000 14 787 803 10.1046/j.1468-2982.2000.00118.x 11167909

[B5] Bahra A May A Goadsby PJ Cluster headache: a prospective clinical study with diagnostic implications Neurology 2002 14 354 361 10.1212/WNL.58.3.354 11839832

[B6] Schurks M Kurth T de Jesus J Jonjic M Rosskopf D Diener HC Cluster headache: clinical presentation, lifestyle features, and medical treatment Headache 2006 14 1246 1254 10.1111/j.1526-4610.2006.00534.x 16942468

[B7] Gaul C Christmann N Schröder D Weber R Shanib H Diener HC Holle D Differences in clinical characteristics and frequency of accompanying migraine features in episodic and chronic cluster headache Cephalalgia 2012 14 571 577 10.1177/0333102412444012 22529192

[B8] Sjaastad O Bakketeig LS Cluster headache prevalence. Vaga study of headache epidemiology Cephalalgia 2003 14 528 533 10.1046/j.1468-2982.2003.00585.x 12950378

[B9] Torelli P Beghi E Manzoni GC Cluster headache prevalence in the Italian general population Neurology 2005 14 469 474 10.1212/01.WNL.0000150901.47293.BC 15699377

[B10] Katsarava Z Obermann M Yoon MS Dommes P Kuznetsova J Weimar C Diener HC Prevalence of cluster headache in a population-based sample in Germany Cephalalgia 2007 14 1014 1019 10.1111/j.1468-2982.2007.01380.x 17666085

[B11] Fischera M Marziniak M Gralow I Evers S The incidence and prevalence of cluster headache: A meta-analysis of population-based studies Cephalalgia 2008 14 614 618 10.1111/j.1468-2982.2008.01592.x 18422717

[B12] Broner SW Cohen JM Epidemiology of cluster headache Curr Pain Headache Rep 2009 14 141 146 10.1007/s11916-009-0024-y 19272280

[B13] Rozen TD Fishman RS Cluster headache in the United States of America: demographics, clinical characteristics, triggers, suicidality, and personal burden Headache 2012 14 99 113 10.1111/j.1526-4610.2011.02028.x 22077141

[B14] Lin KH Wang PJ Fuh JL Lu SR Chung CT Tsou HK Wang SJ Cluster headache in the Taiwanese – A clinic-based study Cephalalgia 2004 14 631 638 10.1111/j.1468-2982.2003.00721.x 15265051

[B15] Imai N Yagi N Kuroda R Konishi T Serizawa M Kobari M Clinical profile of cluster headaches in Japan: Low prevalence of chronic cluster headache, and uncoupling of sense and behaviour of restlessness Cephalalgia 2011 14 628 633 10.1177/0333102410391486 21278239

[B16] Yu S Liu R Zhao G Yang X Qiao X Feng J Fang Y Cao X He M Steiner T The prevalence and burden of primary headaches in China: a population-based door-to-door survey Headache 2012 14 582 591 10.1111/j.1526-4610.2011.02061.x 22590713

[B17] Manzoni GC Terzano MG Moretti G Cocchi M Clinical observations on 76 cluster headache cases Eur Neurol 1981 14 88 94 10.1159/000115213 7215402

[B18] Ekbom K Svensson DA Traff H Waldenlind E Age at onset and sex ratio in cluster headache: observations over three decades Cephalalgia 2002 14 94 100 10.1046/j.1468-2982.2002.00318.x 11972575

[B19] Torelli P Cologno D Cademartiri C Manzoni GC Possible predictive factors in the evolution from of episodic to chronic cluster headache Headache 2000 14 798 808 10.1046/j.1526-4610.2000.00145.x 11135023

[B20] Manzoni GC Male preponderance of cluster headache is progressively decreasing over the years Headache 1997 14 588 589 10.1046/j.1526-4610.1997.3709588.x 9385759

[B21] Manzoni GC Gender ratio of cluster headache over the years: a possible role of changes in lifestyle Cephalalgia 1998 14 138 142 10.1046/j.1468-2982.1998.1803138.x 9595206

[B22] Rozen TD Niknam RM Shechter AL Young WB Silberstein SD Cluster headache in women: clinical characteristics and comparison with cluster headache in men J Neurol Neurosurg Psychiatry 2001 14 613 617 10.1136/jnnp.70.5.613 11309454 PMC1737364

[B23] Donnet A Lanteri-Minet M Guegan-Massardier E Mick G Fabre N Géraud G Société Française d'Etude des Migraines et Céphalées (SFEMC) Chronic cluster headache: a French clinical descriptive study J Neurol Neurosurg Psychiatry 2007 14 1354 1358 10.1136/jnnp.2006.112037 17442761 PMC2095607

[B24] Silberstein SD Niknam R Rozen TD Young WB Cluster headache with aura Neurology 2000 14 219 221 10.1212/WNL.54.1.219 10636152

[B25] Rozen TD Cluster headache with aura Curr Pain Headache Rep 2011 14 98 100 10.1007/s11916-010-0168-9 21161447

[B26] Pringsheim T Cluster headache: evidence for a disorder of circadian rhythm and hypothalamic function Can J Neurol Sci 2002 14 33 40 11858532 10.1017/s0317167100001694

[B27] Kudrow L The cylic relationship of natural illumination to cluster period frequency Cephalalgia 1987 14 76 78 3442810 10.1177/03331024870070S623

[B28] Strittmatter M Hamann GF Grauer M Fischer C Blaes F Hoffmann KH Schimrigk K Altered activity of the sympathetic nervous system and changes in the balance of hypophyseal, pituitary and adrenal hormones in patients with cluster headache Neuroreport 1996 14 1229 1234 10.1097/00001756-199605170-00001 8817538

[B29] Hofman MA Zhou JN Swaab DF Suprachiasmatic nucleus of the human brain: an immunocytochemical and morphometric analysis Anat Rec 1996 14 552 562 10.1002/(SICI)1097-0185(199604)244:4<552::AID-AR13>3.0.CO;2-O 8694290

[B30] Holland PR Goadsby PJ Cluster headache, hypothalamus, and orexin Curr Pain Headache Rep 2009 14 147 154 10.1007/s11916-009-0025-x 19272281

[B31] Holle D Obermann M Cluster headache and the hypothalamus: causal relationship or epiphenomenon? Expert Rev Neurother 2011 14 1255 1263 10.1586/ern.11.115 21864072

[B32] Hofman MA Purba JS Swaab DF Annual variations in the vasopressin neuron population of the human suprachiasmatic nucleus Neuroscience 1993 14 1103 1112 10.1016/0306-4522(93)90493-Y 8506022

[B33] Rozen TD Fishman RS Female cluster headache in the United States of America: what are the gender differences? Results from the United States Cluster Headache Survey J Neurol Sci 2012 14 17 28 10.1016/j.jns.2012.03.006 22482825

